# Direct Full-Length RNA Sequencing Reveals an Important Role of Epigenetics During Sexual Reversal in Chinese Soft-Shelled Turtle

**DOI:** 10.3389/fcell.2022.876045

**Published:** 2022-03-25

**Authors:** Tong Zhou, Guobin Chen, Meng Chen, Yubin Wang, Guiwei Zou, Hongwei Liang

**Affiliations:** ^1^ Yangtze River Fisheries Research Institute, Chinese Academy of Fisheries Science, Wuhan, China; ^2^ College of Fisheries and Life Science, Shanghai Ocean University, Shanghai, China

**Keywords:** RNA methylation, nanopore sequencing, epigenetics, chinese soft-shelled turtle, sexual reversal process

## Abstract

Sex dimorphism is a key feature of Chinese soft-shelled turtle (*Pelodiscus sinensis*). The males (M) have higher econosmic value than females (F) due to wider calipash and faster growth. Exogenous hormones like estradiol and methyltestosterone can induce sexual reversal to form new phenotypes (pseudo-female, PF; pseudo-male, PM) without changing the genotype. The possibility of inducing sexual reversal is particularly important in aquaculture breeding, but the underlying biological mechanisms remain unclear. Here we applied a direct RNA sequencing method with ultralong reads using Oxford Nanopore Technologies to study the transcriptome complexity in *P. sinensis*. Nanopore sequencing of the four gender types (M, F, PF, and PM) showed that the distribution of read length and gene expression was more similar between same-sex phenotypes than same-sex genotypes. Compared to turtles with an M phenotype, alternative splicing was more pronounced in F turtles, especially at alternative 3′ splice sites, alternative 5′ splice sites, and alternative first exons. Furthermore, the two RNA methylation modifications m5C and m6A were differentially distributed across gender phenotypes, with the M type having more modification sites in coding sequence regions, but fewer modification sites in 3′UTR regions. Quantitative analysis of enriched m6A RNAs revealed that the N6-methylated levels of *Odf2*, *Pacs2*, and *Ak1* were significantly higher in M phenotype individuals, while the N6-methylated levels of *Ube2o* were reduced after sexual reversal from both M and F phenotypes. Taken together, these findings reveal an important role of epigenetics during sexual reversal in Chinese soft-shelled turtles.

## Introduction

Chinese soft-shelled turtle, an economically valuable aquatic animal, exhibits obvious sexual dimorphism (Liang et al., 2018; Ma et al., 2021; Nagahama et al., 2021). Male individuals are characterized by wider calipash and faster growth rate, and are more popular in the market (Liang et al., 2019a; Zhou et al., 2021). The mechanism of sex determination in the Chinese soft-shelled turtle is genotypic sex determination (GSD), not temperature-dependent sex determination (TSD), and follows the ZZ/ZW chromosomal system where females have a pair of dissimilar ZW micro-sex chromosomes and males have two similar ZZ chromosomes (Kawai et al., 2007; Kawagoshi et al., 2009).

Exogenous hormones can induce sexual reversal in Chinese soft-shelled turtle during the period of sex differentiation (Schulz et al., 2007; Liang et al., 2019b). The balance between estrogens and androgens determines the direction of gonadal development in vertebrates (Ma et al., 2021). Exogenous hormones such as estradiol can induce the transformation from a male phenotype into a pseudo-female (PF) phenotype without changing the genetic gender. The transition from male to female is generally referred to as the male reversal process, while the transition from female to male is called the female reversal process. Male reversal of *P. sinensis* is of great significance to the aquaculture industry because a sexually mature PF can be crossed with a genetic male (ZZ) to obtain all-male offspring (Zhou et al., 2021). However, the biological underpinnings of sexual reversal in *Pelodiscus sinensis* are still little explored.

Transcriptome analysis is an effective tool for gaining insights into biological processes (Kukurba and Montgomery, 2015). Identification and quantification of various transcripts can improve our understanding of the gene functions and the complex mechanisms involved in sex control and sex differentiation, but it remains a challenging task (Li et al., 2020). Previous studies have typically used short-read (<200 nt) RNA sequencing (RNA-seq) followed by reassembly, making it difficult to sequence repetitive regions, resolve large structural variations and homologous gene segments, and differentiate isoforms.

An alternative approach is the long-range direct RNA-seq technology offered by Oxford Nanopore Technologies (ONT) which has no theoretical upper limit for read length and can sequence transcripts from end to end at a single-molecule level (Jenjaroenpun et al., 2018; Lubuta et al., 2019). ONT sequencing is a new generation of single-molecule real-time electrical signal sequencing technology based on nanopores, and its sequencing principles are the same on all platforms. The DNA/RNA double strand is led by a motor protein to bind to the nanopore embedded in a biological membrane. Under the action of the voltage difference between the two sides of the biological membrane, the DNA/RNA strand passes through the nanopore channel at a certain rate. Because of the differences in the chemical properties of different bases in the DNA/RNA chain, when a single base passes through the nanopore channel, it will cause specific changes to the electrical current that flows though the nanopore. These electrical disruptions are then decoded using base calling algorithms to determine the DNA/RNA sequence in real time (Lu et al., 2016; Karamitros and Magiorkinis, 2018; Dumschott et al., 2020).

This technology overcomes many of the shortcomings of traditional RNA-seq techniques as it allows the direct sequencing of RNA transcripts without the need for inverse transcription and amplification (Kono and Arakawa, 2019). The long sequencing reads provided by this method offer a promising tool for the detection of alternative splicing, fusion genes and novel isoforms as well as the accurate characterization of methylation modification sites (Roach et al., 2020). Compared to standard transcriptome analysis, direct RNA-seq is better suited to identify novel splicing isoforms (Li et al., 2020). Nanopore-based sequencing methods have been used to annotate transcriptome structures in a variety of organisms ranging from the relatively simple *Saccharomyces cerevisiae* to complex human cell lines (Byrne et al., 2017; Garalde et al., 2018; Bayega et al., 2021). Direct DNA sequencing can unlock a wealth of information, such as the nuances of alternative splicing, and thereby contribute to the understanding of molecular mechanisms (Garalde et al., 2018). Many novel alternative splicing events continue to be discovered in a variety of biological processes including sexual differentiation and gonadal development (Ule and Blencowe, 2019).

Epigenetics is the study of heritable changes in gene expression that are not the result of alterations in the nucleotide sequence of the gene (Ladd-Acosta and Fallin, 2015; Kaliman, 2019). Epigenetic changes are defined as heritable cellular information which are not genetic and can be transmitted through cell division (Wu et al., 2015). RNA methylation modification is an important branch of epigenetics (Wang and Wang, 2015). N6-methyladenosine (m6A) and C5-methylcytidine (m5C) are two common RNA post-transcriptional modifications in eukaryotes. RNA methylation plays an important role in regulating gene expression and in processes related to editing, stability and degradation of transcripts. In particular, dynamic methylation of adenine and cytosine is essential for cells to adapt to their environment and for the development of complex organisms from a single cell (Traube and Carell, 2017; Yang et al., 2017; Yang, Y. et al., 2017). For example, m6A can regulate the oocyte-to-embryo transition and the reprogramming of somatic cells into induced pluripotent stem cells (Yu et al., 2021). Wang et al. found that the presence of sex-specific RNA modifications may be involved in the regulation of gonadal development and gametogenesis in *Paralichthys olivaceus* (Wang et al., 2020). In the case of m5C, research has shown that m5C methyltransferases are closely associated with various human diseases including cancers (Liu et al., 2017), and m5C modifications are thought to play a role in intracellular and nucleocytoplasmic transport (Sanchez-Vasquez et al., 2018). Although these two RNA methylation modifications play a crucial role in the growth of animals, there are few reports on the epigenetic relationship between RNA methylation modifications and the sexual reversal of Chinese soft-shelled turtles.

In this study, we employed the Nanopore long-read direct RNA sequencing platform to investigate the transcriptomic complexity of *P. sinensis* genders. Moreover, we comprehensively analyzed alternative splicing events and different RNA methylation modifications, including m6A and m5C, which revealed an important role of epigenetics in Chinese soft-shelled turtle gonadal development.

## Materials and Methods

### *P. sinensis* Sample Collection

Female and male *P. sinensis* were cultured in ponds, which were located in Anhui Xijia Agricultural Development Co. Ltd. (Bengbu, China). The turtles are kept in natural ponds with commercial feeds (Jinjia, China) three times a day. The stocking density of juvenile turtles is about 4/m^2^. The transition to PF or PM was induced at the embryonic stage 15th day before sex differentiation by estradiol (E2) or methyltestosterone (MT). We injected 5 µL of 10 mg/mL E2 (E110147, Aladdin, shanghai, China) or MT (M830053, Macklin, shanghai, China) into the fertilized eggs whose eggshells were softened by hydrochloric acid (HCl) to induce the sex reversal of Chinese soft-shelled turtle (Zhou et al., 2021; Othman et al., 2022). The gonads of pseudo-female and pseudo-male individuals began to differentiate at the embryonic stage 15th day and mature after 2 years. After the turtles were 5 months old, the physiological gender and genetic gender were identified by the tail and sex specific markers (Liang et al., 2019b). The turtles were judged to be male if the tail exceeded the calipash edge, otherwise it was judged to be a female. The genetic gender was determined by sex-specific markers (4085-f/r, Supplementary Table S4) using PCR. Those individuals with inconsistent physiological gender and genetic gender had a sex reversal. After 2 years of cultivation of the turtles, the female, male, PF and PM individuals which every group contains three turtles were used in this study (Supplementary Figure S1). The gonad tissues were collected for further analysis after MS-222 (Sigma, St. Louis, MO, United States) treatment. The turtles were injected intraperitoneally with 600 mg/kg of buffered MS-222 (Keifer and Zheng, 2017). All samples were stored at −80°C.

### RNA Extraction

Total RNA was isolated using the RNA prep pure kit (Tiangen, China), and DNA was removed with DNase I (Sigma, St. Louis, MO, United States) following the protocol Green and Sambrook, 2019. The RNA quality was assessed using NanoDrop One spectrophotometer (NanoDrop Technologies, Wilmington, DE, United States) and Qubit 3.0 Fluorometer (Life Technologies, Carlsbad, CA, United States).

### Library Preparation and Nanopore Sequencing

Libraries of direct RNA sequencing were constructed following the ONT Direct RNA Sequencing protocol (Lu et al., 2016; Grünberger et al., 2022). Approximately 30 μg total RNA was enriched for mRNA using NEBNext Poly(A) mRNA Magnetic Isolation Module (E7490S).

The Nanopore RTA adapter (SQK-RNA002) was added to the Poly(A) mRNA using T4 DNA Ligase (NEB) at 25°C for 10 min, and then the first strand cDNA was synthesized using SuperScript III reverse transcriptase (1808004, Thermo Fisher Scientific, Waltham, MA, United States) to form RNA/DNA duplexes and relax the secondary structure of RNA. The RNA-cDNA hybrid products were purified using 1.8 × Agencourt RNA Clean XP beads and eluted using 23 µL Nuclease-free water (Promega, Madison, WI, United States).

Sequencing adapters were ligated to the mRNA using T4 DNA ligase (NEB) in a total volume of 40 μL, including 23 µL reverse-transcribed mRNA, 8 µL NEBNext Quick Ligation Reaction Buffer (NEB), 6 µL RNA Adapter (RMX; SQK-RNA002), and 3 µL T4 DNA ligase. Then, Agencourt RNA Clean XP beads were added and eluted using 41 µLElution Buffer, and 1 μL eluted sample was quantified by Qubit. Sequencing libraries (145 µL) were prepared by mixing 40 µL mRNA with sequencing adapters, 75 µL RRB (SQK-RNA002), and 30 µL Nuclease-free water. The libraries were loaded on ONT R9.4 flowcells and sequenced according to the standard protocol using the Nanopore PromethION sequencer (Oxford Nanopore Technologies, Oxford, United Kingdom) for 48 h. Three independent biological replicates were sequenced. The base calling was performed concurrent with sequencing based on the Albacore, which is integrated within the MinION software (MinKNOW, v1.10.23). Only “pass” reads were used for subsequent analyses.

### Read Filtering

The reads were filtered with the Miniknow default cutoff of the adaptors, a minimum average q-score of 7 and the length <50 bp. And the data were written out in the FASTQ format using NanoFilt (version:2.6.0). Then the effective reads were further analyzed.

### Mapping and Qualitative Analysis

The clean reads were mapped to the Chinese soft-shelled turtle genome (GCF_000230,535.1) using Minimap2. The parameters were set as “-ax splice -uf -k14”. The blasted transcripts were classified into “unmapped,” “Forward mapped,” and “Reverse mapped”.

### Alternative Splicing Analysis

We utilized the SUPPA2 method (https://github.com/comprna/SUPPA) to analyze the alternative splicing of each individual. In total, seven types of alternative splicing events were detected, including skipping exon, mutually exclusive exons, alternative 5′ splice-site, alternative 3’ splice-site, retained intron, alternative first exon and alternative last exon. The analysis of alternative splicing events was carried out by SUPPA2.

### Characterization of Poly(A) Tail Length

Poly(A) tail lengths of each read were calculated from raw signals using the program nanopolish with “polya” module (version 0.12.5) (Loman and Watson, 2015). Only poly(A) tail length estimates marked as “PASS” were considered for downstream analyses.

### Identification of RNA Methylation Sites

The modifications sites of RNA sequences were identified with Tombo version 1.5 with alternative model. The models of “5mC” and “*de novo*” were implemented separately to detect possible modifications in each read. The score on each site indicated the fraction of a possible modification on a given site. For plotting the modification coverage along the gene body, the modification coverage was normalized for each isoform using a “w0” method with a bin size of 5 nt with EnrichedHeatmap (Gu et al., 2018). Only the isoforms with both 5′ and 3′UTRs longer than 50 nt were used in the calculation. The identification of m6A sites was carried out based on the *de novo* of Tombo (Lorenz et al., 2020).

### Enrichment of m6A RNA From Total RNAs

The EpiQuik CUT&RUN m6A RNA Enrichment kit (EpiGentek, P-9018) was used to hatch the m6A RNAs from total RNAs which were extracted from gonad tissues from the different samples. In this reaction, RNA sequences in both ends of the target m6A-containing regions are cleaved/removed, and the target m6A-containing fragments are pulled down using a beads-bound m6A capture antibody. The enriched RNA is then released, purified, and eluted. The eluted RNAs were used for further quantitative analyses.

The subsequent quantification of cDNA products was performed as described previously (Zhou et al., 2021). The primers for target gene qRT-PCR are provided in Supplementary Table S4. Relative m6A mRNA expression levels were calculated by the 2−(∆∆Ct) method (Schmittgen, 2008).

### Statistical Analysis

All the experimental data from at least three independent experiments were analyzed using GraphPad Prism 7.0 software (San Diego, CA, United States) and were presented as mean ± SD. Student’s t-test or One-Way ANOVA were performed to compare the pairwise differences between groups.

## Results

### Statistics of Direct RNA Sequencing Data of Different Samples

**FIGURE 1:**
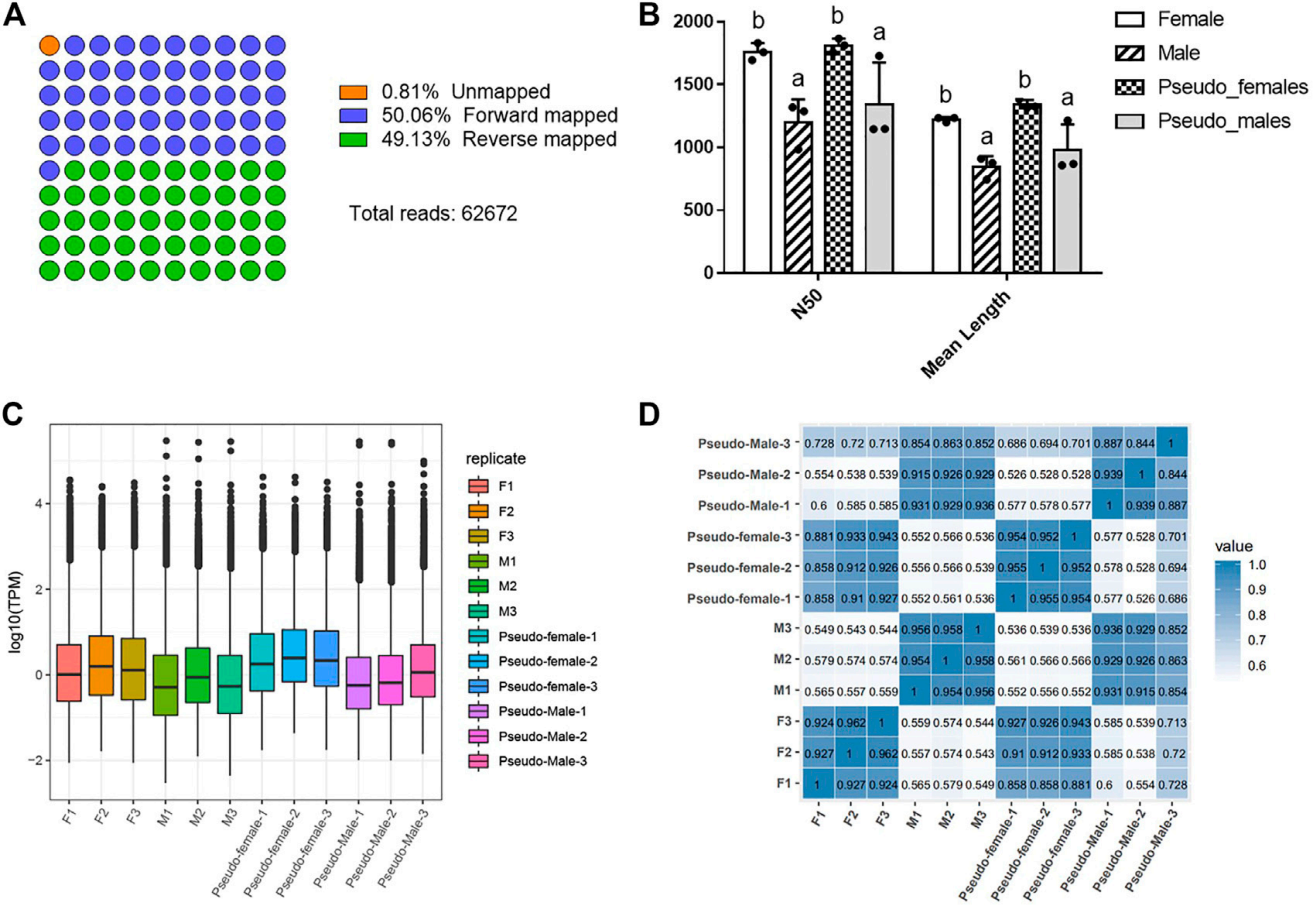
Statistics of direct RNA sequencing data of different samples. **(A)** The mapped diagram of sequencing data. **(B)** The histogram of the distribution of N50 and mean length. Data were expressed as mean ± SD. Different superscripts indicate significant difference (*p* < 0.05). **(C)** The TPM box plot of different samples. **(D)** The correlation analysis on the different samples. F1: Female-1, F2: Female-2, F3: Female-3, M1: Male-1, M2: Male-2, M3: Male-3, PF1: Pseudo-female-1, PF2: Pseudo-female-2, PF3: Pseudo-female-3, PM1: Pseudo-male-1, PM2: Pseudo-male-2, PM3: Pseudo-male-3.

To assess the quality of the RNA sequencing data, we identified basic data indicators such as the read number, the assembly quality metric N50, and mean length of sequencing reads, and compared these between different physiological and genetic genders (Figure 1). Since the raw sequencing data may contain low-quality sequences and adapter sequences, we filtered the data to obtain clean reads and thereby ensure the reliability of the analysis results. After filtering, 62,672 clean reads were mapped to the Chinese soft-shelled turtle genome (version 1.1) using Minimap2 (v.2.17-r941). The results showed that forward mapped reads and reverse mapped reads occupied 99.19% of total clean reads (Figure 1A, Supplementary Table S1). A histogram displaying the distribution of read lengths across quality passing sequences is shown in Supplementary Figure S2. For each gender, we obtained >1.5 million reads with an average read length of 1,223, 844, 1,341, 979 for female (F), male (M), PF and PM, respectively. The N50 statistic of the four gender types (F, M, PF, and PM) were 1760, 1,197, 1811 and 1,339. The N50 and mean length of PM were close to those of M while far from those of F. On the contrary, the N50 and mean length of PF were close to those of F but far from those of M. These characteristics of basic data among four types of Chinese soft-shelled turtle showed that the changes brought about by sex reversal (Figure 1B, Supplementary Table S2). TPM box plot showed the median log2 (TPM) values for all transcripts in each group were different and the values of M and PM were lower than F and PF (Figure 1C). We conducted correlation analysis on the different samples, and found a high degree of correlation between most of them, meaning that they met the basic requirements for further analysis (Figure 1D). In general, the sequencing data had high depth and confidence, and the M and PM groups were different from the F and PF in read length and the overall expression level of transcripts.

### Structure Analysis of *P. sinensis* Genders

**FIGURE 2:**
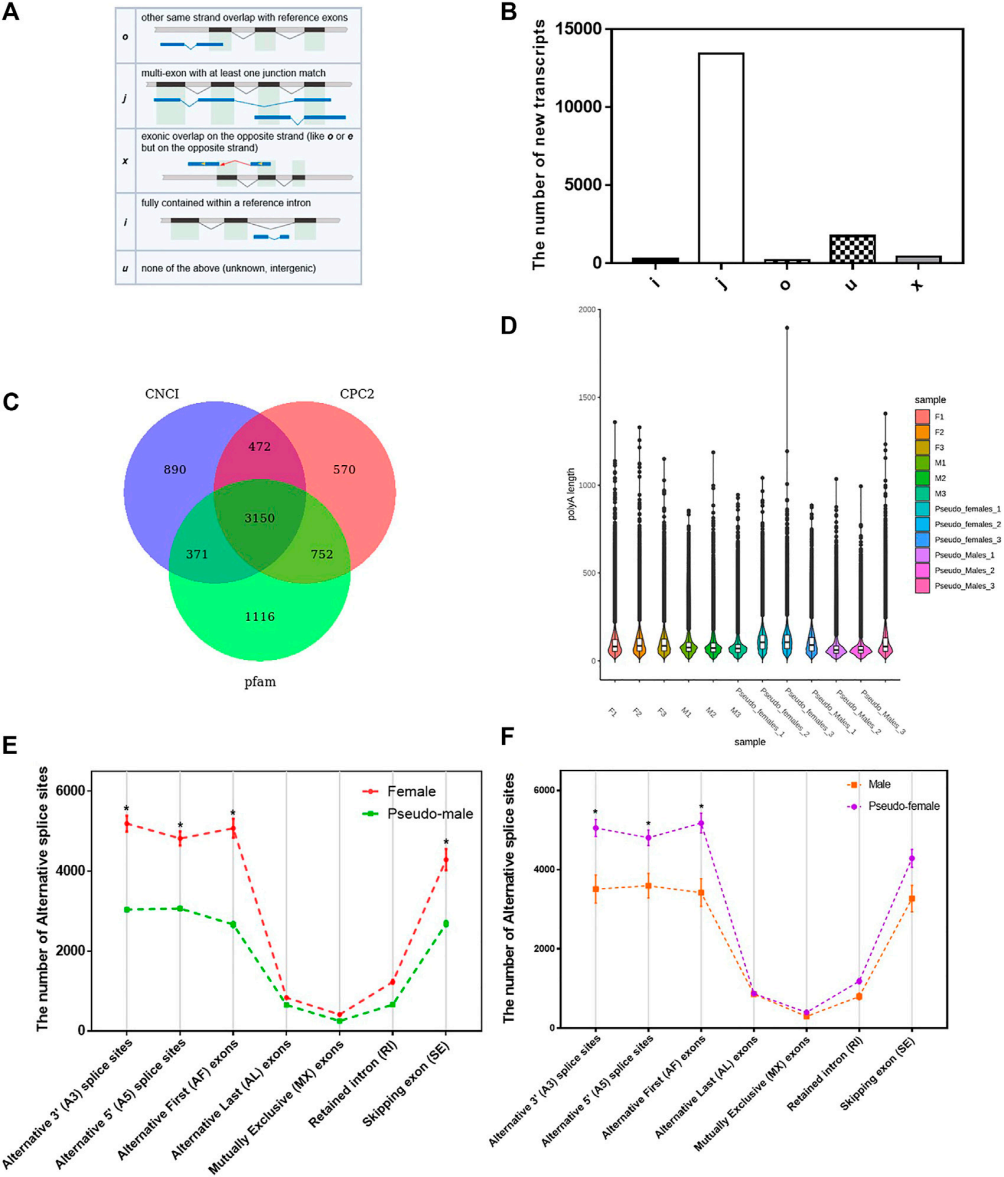
Structure analysis of *P. sinensis* genders. **(A)** The structure of 5 new transcripts types. **(B)** The number of 5 new transcripts types. **(C)** The Wayne analysis of the number of long noncoding RNA transcripts identified by three software (CNC1, CPC2, and Pfam). **(D)** The distribution of the length of ploy(A) tails of different samples. **(E)** The number of alternative splice sites of females and pseudo-males. **(F)** The number of alternative splice sites of males and pseudo-females. Data were expressed as mean ± SD. *p < 0.05.

The annotations of the selected reference genomes are often not accurate enough due to software and data limitations, making it necessary to optimize the structure of the original annotated transcripts. We used gffcompare to compare the annotated transcripts with the known transcripts of the genome, to find new transcripts, and to supplement transcript annotations. In total, we identified 5 new transcript types (O, J, X, I, and U) containing 16,077 transcripts (Figures 2A,B) Samples correlation matrix revealed that there was good agreement both between and within the sample groups (Supplementary Figure S3A).

Long noncoding RNA is a type of RNA molecule whose transcript length exceeds 200 nt and does not encode a protein. We used three software (CNC1, CPC2, Pfam) to analyze the number of long noncoding RNA transcripts (Figure 2C). Differentially expressed long noncoding RNAs in M vs. PF and F vs. PM were enriched for many biological processes such as cellular process, single-organism process and biological regulation (Supplementary Figures S3B,C). Poly(A) tails are known regulators of mRNA stability and intracellular transport. Since the length of ploy(A) tails can affect the translation efficiency, the analysis of their length can uncover the dynamic processes of mRNA and protein translation. Based on the direct mRNA sequencing data, the global poly(A) profiles were analyzed, and length distributions were found to be different between the four gender populations but consistent within each group (Figure 2D). We next analyzed the alternative splicing types in each sample and counted the number of transcripts in each type. As shown in Figure 2E, the number of alternative splicing sites at positions such as alternative 3′ splice site (A3), alternative 5’ splice site (A5) and alternative first exons (AF) had significant differences between F and PM, which the number of alternative splicing sites was obviously reduced in the sex reversal process from F to PM. During the sex reversal process from M to PF, the number of alternative splicing sites at those positions was significantly increased (Figure 2F).

### Differential Gene Expression Analysis Between Gender Types

**FIGURE 3:**
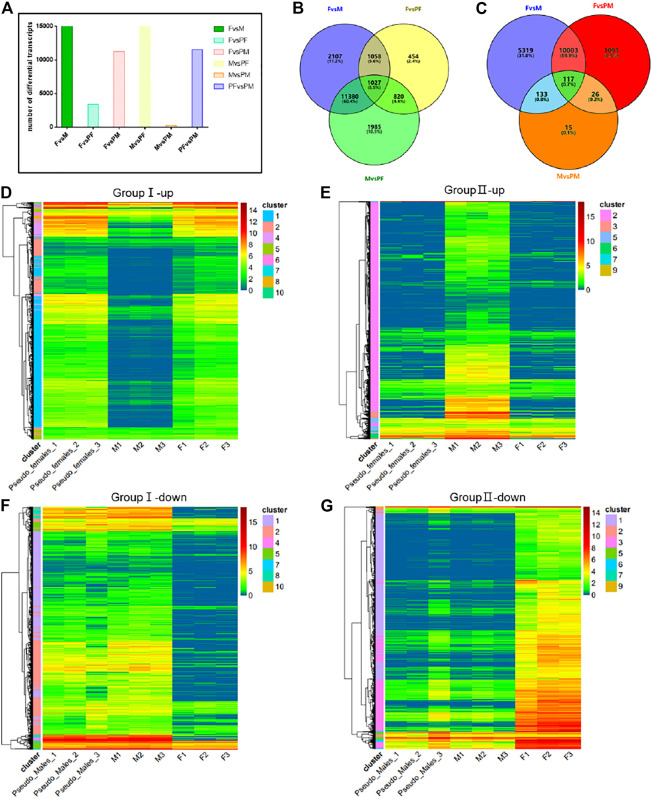
Differential gene expression analysis between gender types. **(A)** The number of differential transcripts in pairs. **(B)** The Wayne analysis of differential genes among females, males and pseudo-females. **(C)** The Wayne analysis of differential genes among females, males and pseudo-males. **(D)** The cluster analysis of differential up-regulated genes of group I (i.e., F, M, and PF). **(E)** The cluster analysis of differential up-regulated genes of group II (i.e., F, M, and PM). **(F)** The cluster analysis of differential down-regulated genes of group I (i.e., F, M, and PF). **(G)** The cluster analysis of differential down-regulated genes of group II (i.e., F, M, and PM).

To further evaluate the differences between the four *P. sinensis* genders, we conducted pairwise differential gene expression analyses. Approximately 15,000 differentially expressed genes were identified between M and F or M and PF, while 12,000 differential genes were found between female individuals and male phenotype individuals. The number of differentially expressed genes between the same gender (M and PM; F and PF) was small (Figure 3A). We divided these data into two groups (group I and group II) for analysis. Group I (i.e., F, M, PF) consisted of the differentially expressed genes related to the gender reversal process from male to pseudo-female, and group II (i.e., F, M, PM) consisted of the differential genes related to the F to PM transition. The numbers of differentially expressed genes in these two groups as determined by Wayne analysis are shown in Figures 3B,C.

We performed cluster analysis on the differential genes in the two groups, and the up-regulated and down-regulated genes clusters identified for group I are shown in Figures 3D,F, while group II results are shown in 3E and 3G. All the differentially expressed genes were clustered into 10 categories, and among the up-regulated genes, those of the first category constituted the largest proportion, whereas the genes of the second category ranked first among the down-regulated differential genes in group I. GO enrichment analysis revealed that a large proportion of the up-regulated genes are involved in processes related to the endosome membrane, cellular calcium ion homeostasis and autophagosome (Supplementary Figure S4A). KEGG enrichment analysis showed that several signal pathways such as microRNA in cancer, protein processing in ER and influenza A were associated with the transition from M individuals to PF individuals (Supplementary Figure S4B). GO enrichment analysis of the second category among down-regulated differential genes showed processes related to motile cilium, axoneme and microtubule-based movement were significantly reduced during sex reversal (Supplementary Figure S4C). The down-regulated differential genes were mainly concentrated in the purine metabolism and Huntington disease signaling pathways (Supplementary Figure S4D). The differential genes of group II were likewise clustered into 10 categories, and cluster results indicated that the majority of up-regulated and down-regulated differential genes were in the first category (Figures 3E,G). GO and KEGG enrichment analysis results showed that the differential genes of the first category were mainly enriched in vesicle-mediated transport, motile cilium and multiple signaling pathways such as hippo signaling pathway and glycerophospholipid metabolism (Supplementary Figures S4E,F).

### Methylation Analysis of *P. sinensis* Gender Types

**FIGURE 4:**
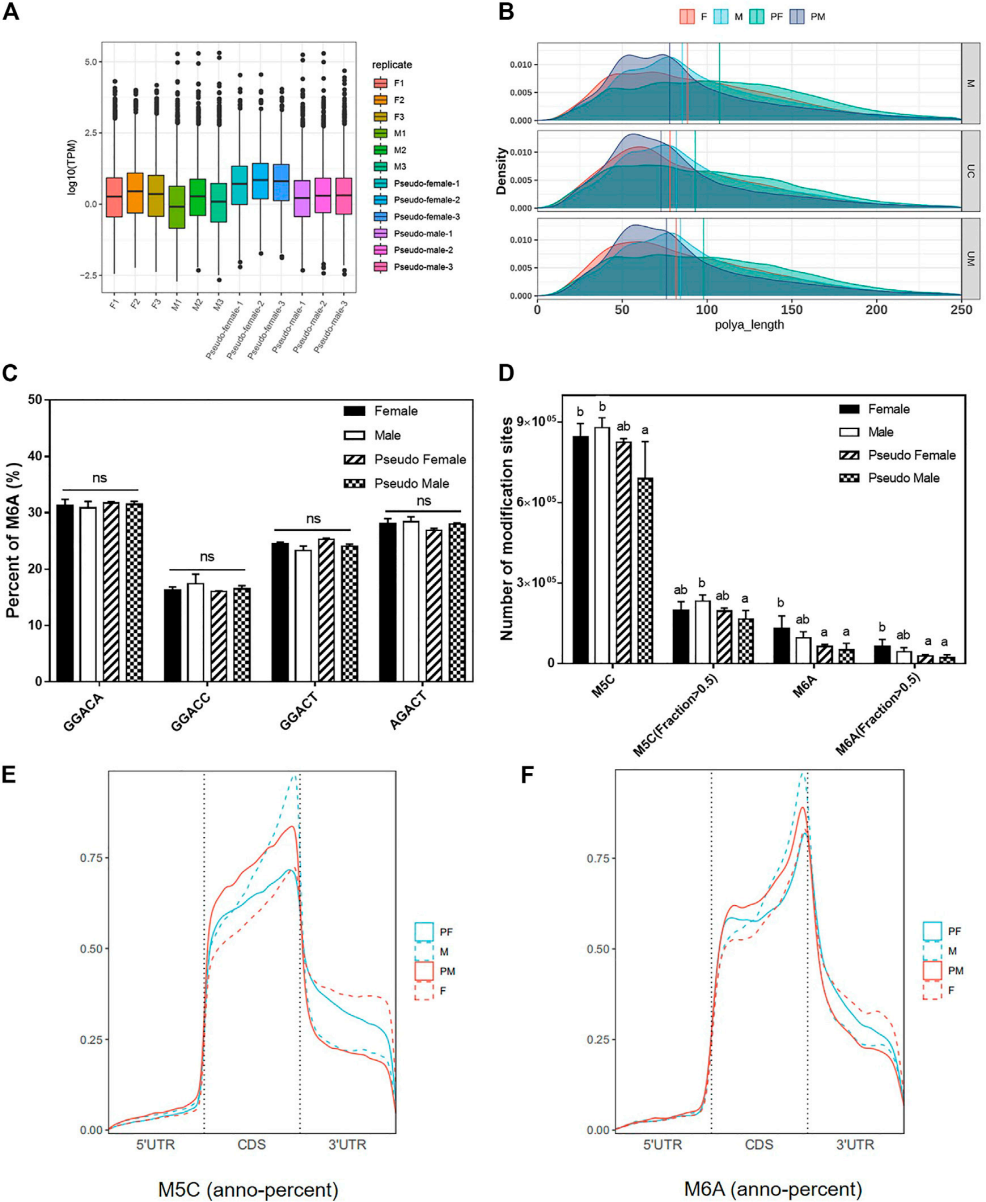
Methylation analysis of *P. sinensis* gender types. **(A)** The distribution and overall expression levels of transcripts with m6A modifications of different samples. **(B)** The correlation analysis between poly(A) length and transcripts with m6A modification of different samples. **(C)** The percentage of different m6A methylation types of *P. sinensis* gender types. **(D)** The number of m5C and m6A modification sites of *P. sinensis* gender types. **(E)** The percentage of m5C methylation annotation in different positions. **(F)** The percentage of m6A methylation annotation in different positions. Data were expressed as mean ± SD. Different superscripts indicate significant difference (*p* < 0.05). ns, no significant difference.

The distribution and overall expression levels of transcripts with m6A modifications are shown in Figure 4A. The expression levels of the F phenotype were significantly higher than the expression level of M phenotype. Correlation analysis between polyA length and transcripts with m6A modification indicated a significant difference between female phenotype samples (F and PF) and male phenotype samples (M and PM; Figure 4B). We identified four different sequences of five bases each which are known m6A methylation sites, and compared the percentage of these sequences that harbored a m6A methylation. We found no significant difference between gender groups (Figure 4C). Then, we counted the total number of methylation modification sites including m5C and m6A, and found that the individuals induced by exogenous hormones (E2 and MT) had fewer methylation modification sites (Figure 4D).

Methylated gene regions (m5C and m6A) were annotated based on the reference genome of soft-shelled turtle. The results of anno-percent (Methylation annotation percentage) of m5C showed that the location of methylated modification sites had changed from CDS regions to 3′UTR regions during the process of sex reversal from male to pseudo-female. However, the location of many methylated modification sites (m5C) had changed from 3′UTR regions to CDS regions during female transition (Figure 4E). As with m5C modifications, the position of m6A modification sites also changed. Compared to female phenotype individuals, male phenotype individuals had more modification sites in the CDS regions and fewer modification sites in 3′UTR regions (Figure 4F). These results suggest that the PF is more similar to the F with respect to methylation modifications, while the PM is more similar to the M.

### Analysis of m6A Modification Genes Across Gender Types

**FIGURE 5:**
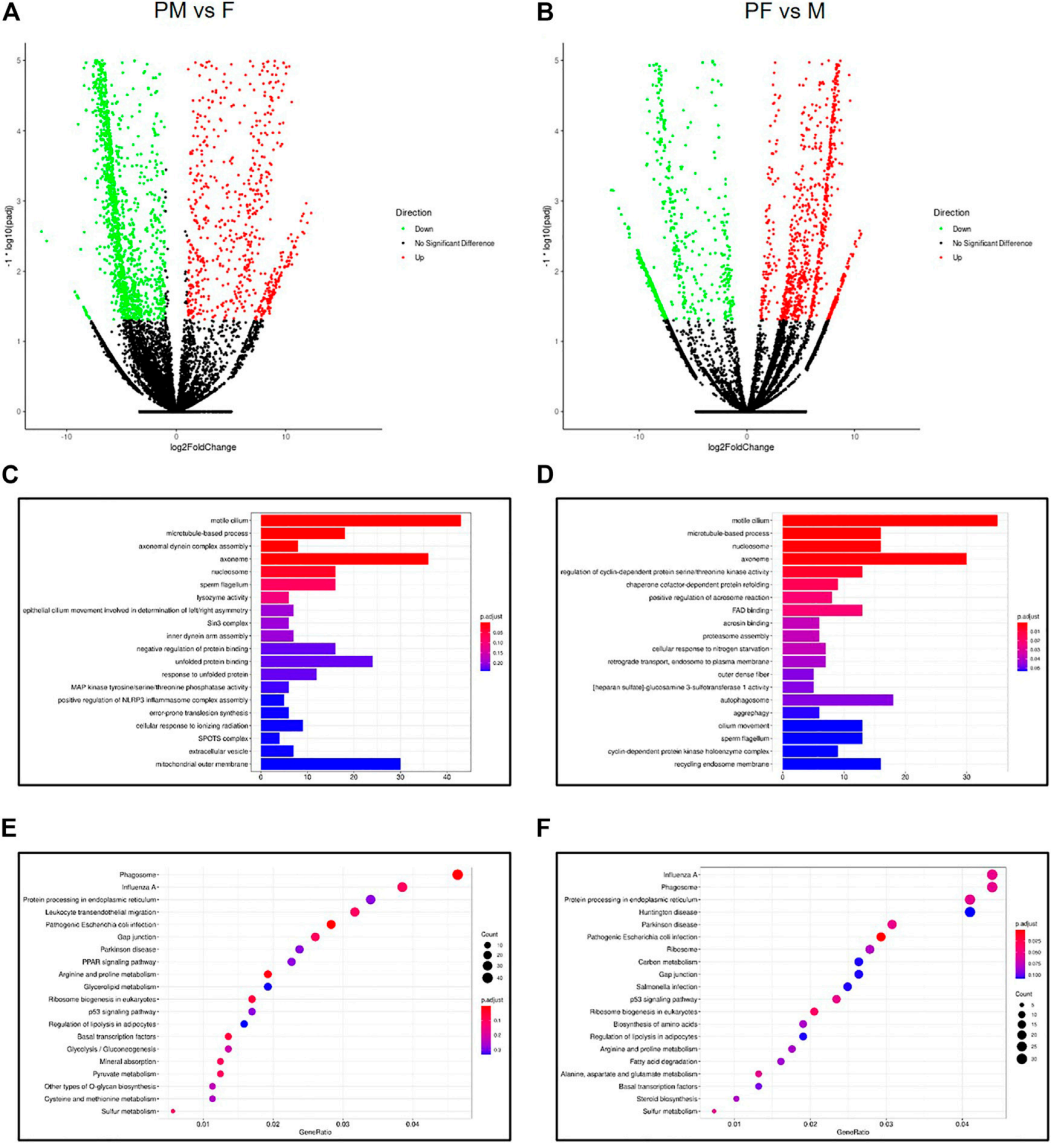
Analysis of m6A modification genes across gender types. **(A)** The Volcano map of differential m6A methylation genes between PM and F. **(B)** The Volcano map of differential m6A methylation genes between PF and M. **(C)** The GO enrichment analysis of differential m6A methylation genes between PM and F. **(D)** The GO enrichment analysis of differential m6A methylation genes between PF and M. **(E)** The KEGG enrichment analysis of differential m6A methylation genes between PM and F. **(F)** The KEGG enrichment analysis of differential m6A methylation genes between PF and M.

To further analyze the differentially expressed m6A modification genes among the four gender types, we conducted two sets of differential expression analyses: F vs. PM and PF vs. M (Figures 5A,B). In the first analysis, we identified 1,038 up-regulated and 1743 down-regulated m6A-modified genes, while in the second set we identified 1,279 up-regulated and 880 down-regulated m6A-modified genes.

GO enrichment analysis of the genes in the first set showed enrichment for the GO terms motile cilium, axoneme and mitochondrial outer membrane, indicating that these processes play an important role in the transition from F to PM (Figure 5C). In addition, KEGG enrichment analysis of the same set of genes identified several significant pathways such as phagosome, influenza A and protein processing in ER (Figure 5E). Likewise, GO and KEGG analyses of the differentially expressed genes in the PF vs. M set showed enrichment for many of the same GO terms, including motile cilium, axoneme and autophagosome (Figure 5D), as well as many of the same KEGG pathways, such as phagosome, influenza A and protein processing in ER (Figure 5F). These findings suggest that similar biological processes drive gender reversal in either direction.

### The Chromosomal Location of Methylated Modification Sites

**FIGURE 6:**
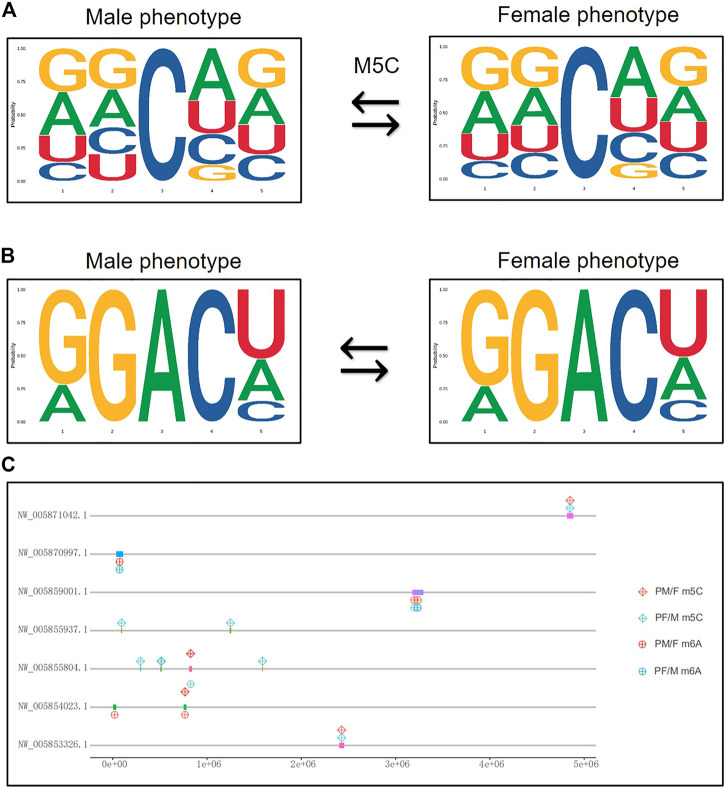
The chromosomal location of methylated modification sites. **(A)** The genetic features of the 5 bp long m5C methylation sites from male phenotype to female phenotype. **(B)** The genetic features of the 5 bp long m6A methylation sites from male phenotype to female phenotype. The abscissa represents the number of bases at the methylation site, the total height of each position is the sequence conservation of the bases, and the height of the base signal represents the relative frequency of the base at that position. **(C)** The chromosomal location of differential methylated gens both in m5C and m6A related sexual reversal process.

The genetic features of the 5 bp long m5C and m6A methylation sites were analyzed using the MEME Suite (Figures 6A,B). The results showed that the position of the second base in the m5C methylation site changed between male phenotype and female phenotype, while the m6A sequence features did not change (Figure 6B).

Given that the differential methylated genes seem to play a significant role in the transformation of male and female phenotypes, we analyzed and mapped some crucial genes to their chromosomal positions. Figure 6C shows the location of differential m5C and m6A modification genes that were identified in both sexual reversal pathways. These genes were mainly located on seven chromosomes (NW_005871042.1, NW_005870997.1, NW_005859001.1, NW_005855937.1, NW_005855804.1, NW_005854023.1, and NW_005853326.1), suggesting that these methylated chromosomes may be closely related to gender differentiation in both directions.

### Verification of N6-Methylation Modification Sites of Sex-Related Genes

**FIGURE 7:**
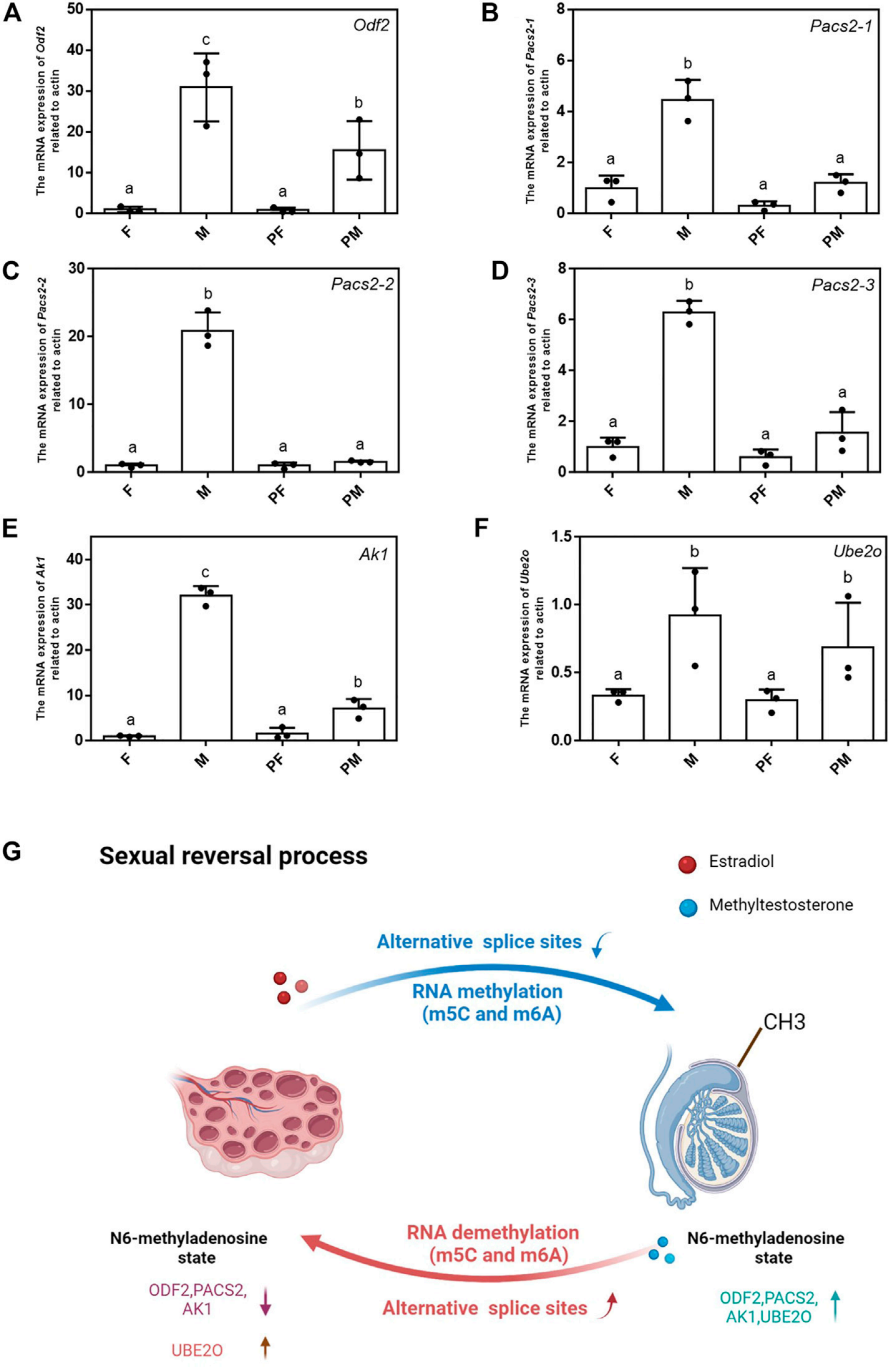
Verification of N6-methylation modification sites of sex-related genes. **(A–F)** The mRNA expression level of *Odf2, Pacs2-1, Pacs2-2, Pacs2-3, Ube2o, Ak1* related to actin among 4 types. Data were expressed as mean ± SD. Different superscripts indicate significant difference (*p* < 0.05). **(G)** The schematic of the changes of two sexual reversal processes.

All the differentially expressed methylated genes located in these chromosomes are listed in Supplementary Table S3. We designed the primers corresponding to these differential genes and verified the expression of N6-methylated fragments. The methylated RNAs were captured by magnetic beads with m6A antibody and were reverse transcribed into cDNA for further analysis (Supplementary Table S4). The quantitative results showed that the N6-methylated levels of *Odf2, Pacs2, Ak1* and *Ube2o* in the transition from M to PF were significantly reduced, while the N6-methylated levels of *Odf2, Pacs2, Ak1* in the transition from F to PM increased and the N6-methylated level of *Ube2o* decreased (Figures 7A–F). The changes in RNA methylation of these genes can play a significant role in the sexual reversal process.

A schematic summarizing the differences between the two sexual reversal ways is shown in Figure 7G. The schematic displays the most important changes in the sexual reversal process, including the number of different alternative splice sites, location of RNA methylations (m5C and m6A) and the N6-methylated levels of critical sex-related genes. Taken together, these results demonstrate that epigenetics plays an indispensable role in the sexual reversal process.

## Discussion

Sex differentiation and differences in vertebrates have always attracted attention (Nagahama et al., 2021). Many animals, including many aquatic animals, show clear gender dimorphism (Dean and Mank, 2014), which has provided an important impetus for new approaches to aquatic breeding. In recent years, the new aquatic species of yellow catfish, Japanese flounder and tilapia were successfully bred based on gender dimorphism (Palaiokostas et al., 2015; Zhang et al., 2016). Chinese soft-shelled turtle is another economically important aquatic animal that displays a high degree of gender dimorphism (Zhang et al., 2018). Despite years of study, our understanding of the biological basis of sexual dimorphism of Chinese soft-shelled turtle remains incomplete. An antagonism between the male and female pathway genes exists in gonads during both sex differentiation and, surprisingly, even as adults, suggesting that in addition to sex-changing fishes, gonochoristic vertebrates like mice maintain some degree of gonadal sexual plasticity into adulthood (Godwin, 2010; Li et al., 2019; Nagahama et al., 2021). Exogenous hormones such as estradiol and methyltestosterone can break the balance between estrogen and androgen, which can change the direction of gonadal differentiation (Balthazart et al., 2009; Carvalho et al., 2018; Toyota et al., 2020). In this study, we used exogenous hormones estradiol (E2) and methyltestosterone (MT) to stimulate the embryos of the Chinese soft-shelled turtle during the gonadal differentiation stage, and obtained PF and PM individuals.

Understanding sexual reversal requires insight into RNA transcription, processing, and modification (Parker et al., 2020). We employed nanopore direct RNA sequencing to examine the transcriptome of four gender types (F, M, PF, and PM) of Chinese soft-shelled turtle with a special focus on RNA methylation (m5C and m6A). Because of the long reads generated by nanopore RNA-seq (>1900 bp in this study), it can overcome some of the limitations of traditional next-generation RNA-seq, and thus reveal the complexity of RNA processing and modification in full-length single molecule reads.

Alternative splicing (AS) has emerged as a key event during gonad development in vertebrate (Domingos et al., 2018; Zhao et al., 2018). In this study, we showed that female turtles had more splicing events than male turtles, and that they were mainly concentrated in alternative 3′ splice sites (A3), alternative 5’ splice sites (A5) and alternative first exons (AF). Changes in alternative splicing events can cause evolutionary alterations in RNA binding proteins and related cis-elements (Ule and Blencowe, 2019). We found that the number of splicing events was significantly increased in female phenotype individuals, and that the related proteins and biological processes of the female pathway were consequently more complex. We speculated that these differential transcripts may have important regulatory effects on gonadal differentiation.

Epigenetics and genetics work together to affect the differentiation direction of the gonads in vertebrates (Gunes et al., 2016; Credendino et al., 2020; Ortega-Recalde et al., 2020). RNA methylation modification is an important type of epigenetics which has attracted much attention in recent years (Fu et al., 2019; Mongan et al., 2019). Methylation of the N (6) position of adenosine (m6A) is a posttranscriptional modification. The changes of RNA methylation sites may influence the progression of several diseases such as cancer (Meyer et al., 2012; Lin et al., 2019). During the process of sexual reversal, we found that the location of several methylation modification sites had changed. The methylation modification sites (m5C and m6A) on 3′UTRs were significantly increased in the transition from M individuals to PF individuals. By contrast, F individuals had strikingly fewer methylation sites on 3′UTRs compared to PM individuals. The changes of methylation sites revealed that the same genes may have different expression patterns in different gender types, even though their genotypes remain unchanged (Zhang et al., 2021). The transition of methylation position between CDS and 3′UTR proved that these changed genes are closely related to the differentiation of gonads.

Several studies have demonstrated an important role of epigenetics in the process of sexual reversal (Shao et al., 2014; Scaduto et al., 2017; Ortega-Recalde et al., 2020). For example, the X chromosome protects against bladder cancer in female mice via a *KDM6A*-dependent epigenetic mechanism (Kaneko and Li, 2018). Shao et al. found that epigenetic regulation plays multiple crucial roles in sexual reversal of tongue sole fish (Shao et al., 2014). However, the connection between RNA methylation and the sexual reversal process is rarely reported. In this study, we compared the RNA methylation levels of the m5C and m6A modifications between the four gender types of Chinese soft-shelled turtle, and identified ∼2,700 methylated genes that were differently expressed between F and PM and ∼2000 genes that were differentially expressed between M and PF. These genes were subsequently mapped to their chromosomal positions (Figure 6C, Supplementary Table S3). The quantitative results showed that the N6-methylated level of these differential methylated genes was significantly higher in male phenotypic individuals than in female phenotypic individuals (Figure 7). Taken together, our findings suggest that the direction of gender differentiation is determined by the degree of RNA methylation. Meanwhile, the sexual reversal induced by exogenous hormones such as estradiol and methyltestosterone will bring the changes of the location of RNA methylation. In summary, epigenetic regulation such as RNA methylation (m5C and m6A) and alternative splicing events play a key and irreplaceable role in the sexual reversal process of Chinese soft-shelled turtles.

## Data Availability

The datasets presented in this study can be found in online repositories. The names of the repository/repositories and accession number(s) can be found below: BioProject, accession number: PRJNA808173.
