# Supplementary material for: Direct Full-Length RNA Sequencing Reveals an Important Role of Epigenetics During Sexual Reversal in Chinese Soft-Shelled Turtle
Source: Front Cell Dev Biol. 2022 Mar 25;10:876045. doi: 10.3389/fcell.2022.876045 (PMC8990255; doi:10.3389/fcell.2022.876045)
Supplement: Supplementary file 1 [file DataSheet1.PDF]

Supplemental materials

Figure S1. The technical route of this study

Figure S2. The histogram of the distribution of read lengths across quality passing sequences.

Figure S3. Bioinformatics analysis of transcripts of different samples.

(A) The correlation matrix of different samples. (B) The GO enrichment analysis of different long non-coding RNAs between males and pseudo-females. (C) The GO enrichment analysis of different long non-coding RNAs between females and pseudo-males.

Figure S4. GO enrichment analysis of group Ⅰ and Ⅱ.

(A) The GO enrichment analysis of cluster 1 of group Ⅰ. (B) The KEGG enrichment analysis of cluster 1 of group Ⅰ. (C) The GO enrichment analysis of cluster 2 of group Ⅰ. (D) The KEGG enrichment analysis of cluster 2 of group Ⅰ. (E) The GO enrichment analysis of cluster 1 of group Ⅱ. (B) The KEGG enrichment analysis of cluster 1 of group Ⅱ.

Supplemental Table 1. The number of sequencing reads of different samples.

Supplemental Table 2. The statistical analysis of sequencing data.

Supplemental Table 3. The differentially expressed methylated genes in sexual reversal process located in chromosomes.

Supplemental Table 4. The primers used in this study.

Figure S1

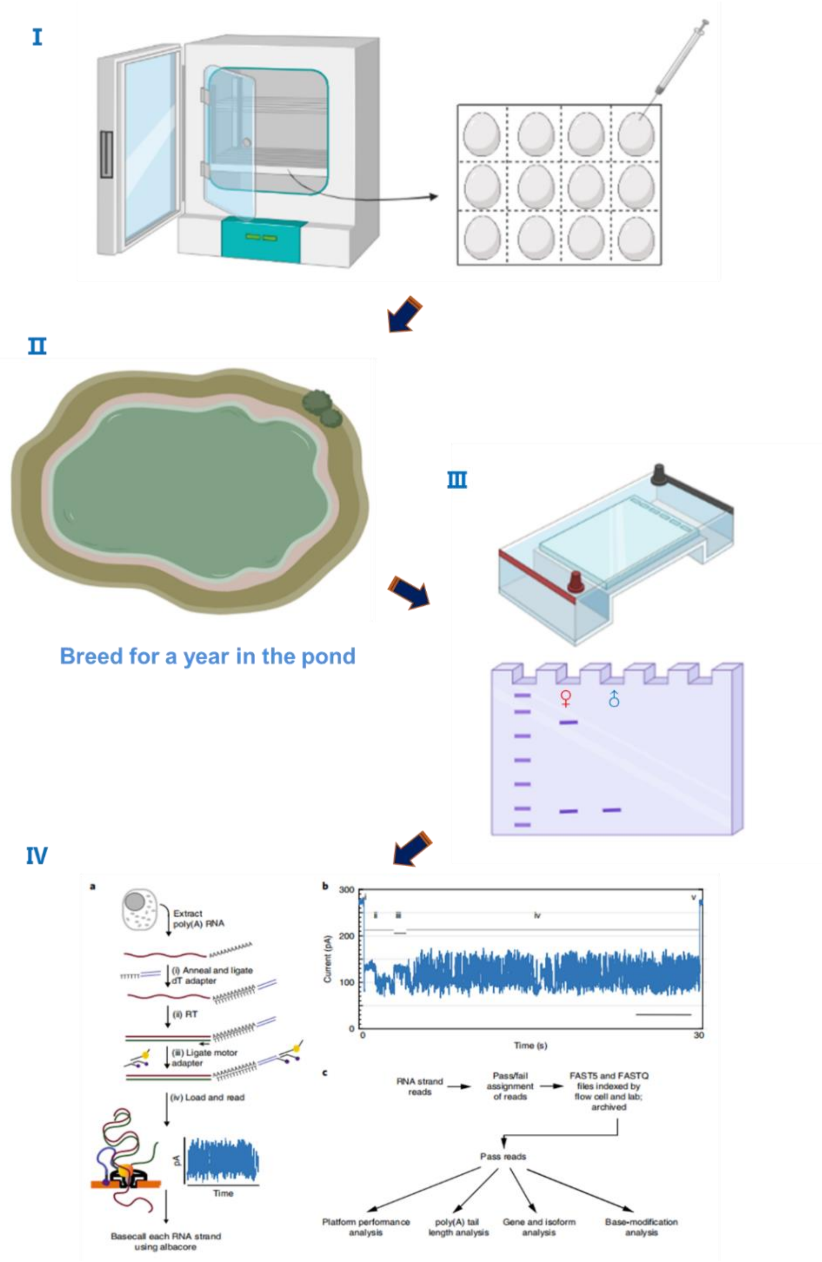

I
II
Breed for a year in the pond
III
♀
♂
IV
a
Extract poly(A) RNA
(i) Anneal and ligate dT adapter
(ii) RT
(iii) Ligate motor adapter
(iv) Load and read
pA
Time
Basecall each RNA strand using albacore
b
Current (pA)
300
200
100
0
Time (s)
30
ii
iii
iv
v
c
RNA strand reads
Pass/fail assignment of reads
FAST5 and FASTQ files indexed by flow cell and lab; archived
Pass reads
Platform performance analysis
poly(A) tail length analysis
Gene and isoform analysis
Base-modification analysis

Figure S2

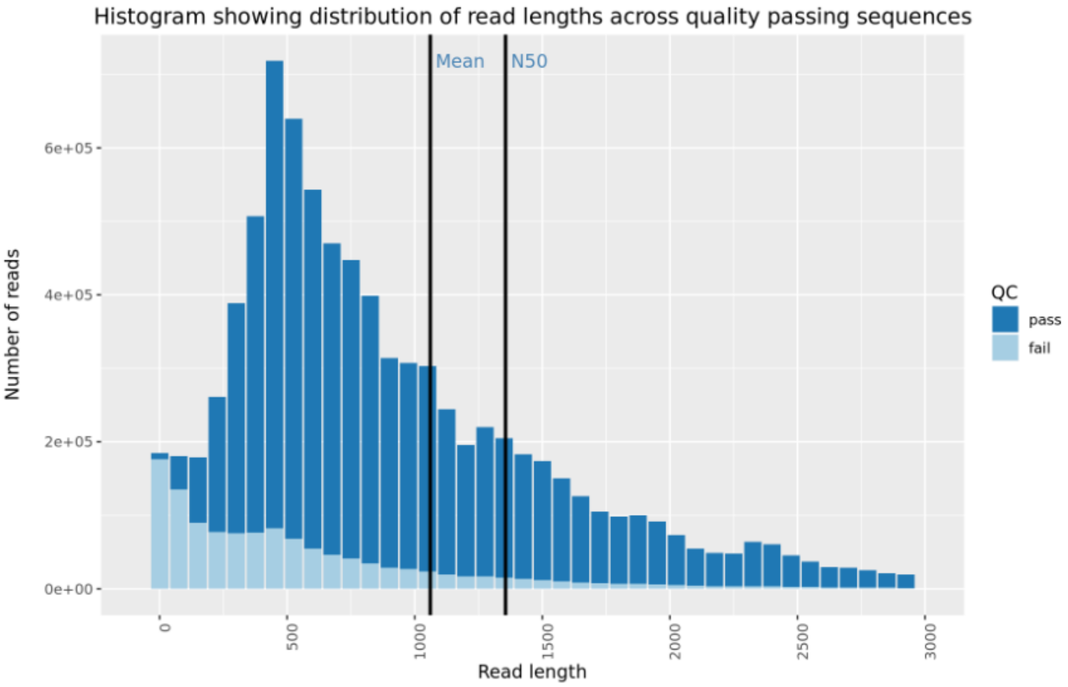
Histogram showing distribution of read lengths across quality passing sequences
Mean
N50
Number of reads
6e+05
4e+05
2e+05
0e+00
0
500
1000
1500
2000
2500
3000
Read length
QC
pass
fail

Figure S3

A

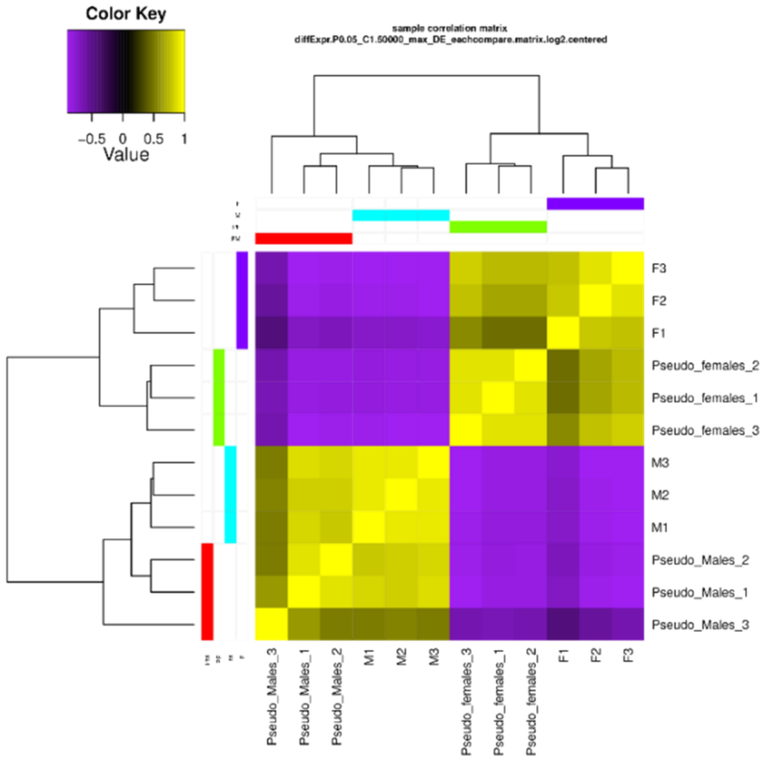

B

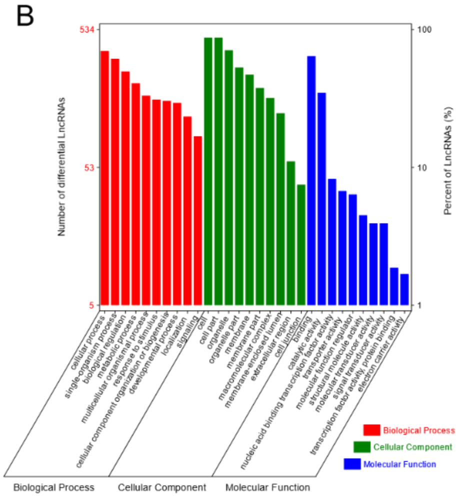

M VS. PF

C

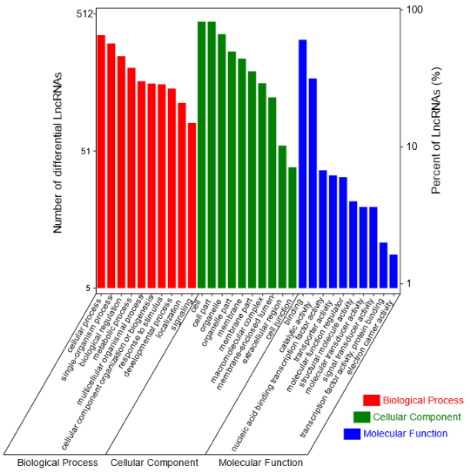

F VS. PM

Figure S4

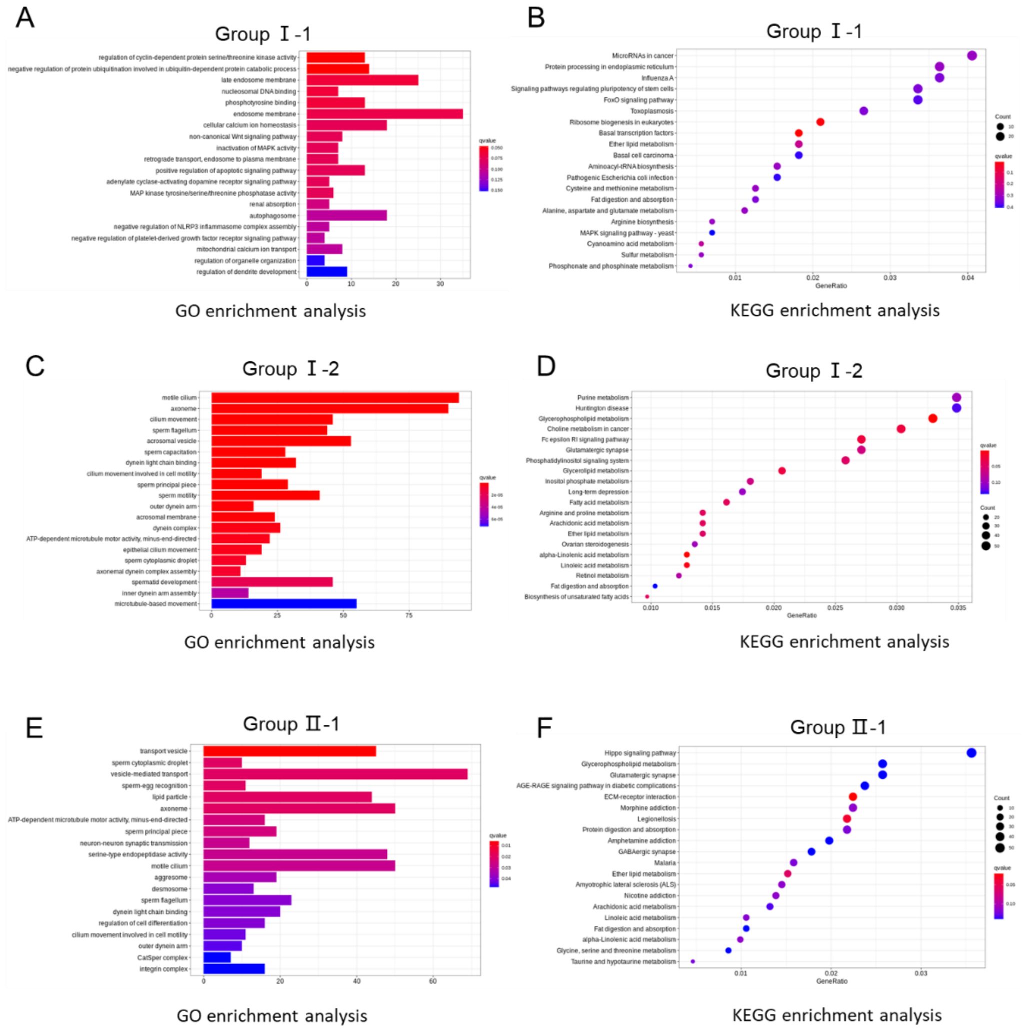
A
Group Ⅰ-1
GO enrichment analysis
B
Group Ⅰ-1
KEGG enrichment analysis
GeneRatio
C
Group Ⅰ-2
GO enrichment analysis
D
Group Ⅰ-2
KEGG enrichment analysis
GeneRatio
E
Group Ⅱ-1
GO enrichment analysis
F
Group Ⅱ-1
KEGG enrichment analysis
GeneRatio

Table S1

Sample	Total reads	mapped reads	Map rate(%)
F1	4,706,568	4,307,585	91.52%
F2	4,427,870	4,077,983	92.10%
F3	5,033,851	4,730,702	93.98%
M1	7,950,368	7,101,701	89.33%
M2	3,579,896	3,244,802	90.64%
M3	6,199,718	5,689,125	91.76%
PM1	2,864,486	2,683,649	93.69%
PM2	2,306,538	2,161,613	93.72%
PM3	2,726,188	2,640,921	96.87%
PF1	2,263,546	2,218,303	98.00%
PF2	1,385,531	1,357,376	97.97%
PF3	2,095,150	2,052,081	97.94%

Table S2

Sample	Seq Num	Mean	Length(bp)	N50(bp)
F1	78,230	2,183.20	19,427	2,897
F2	59,589	2,297.50	15,911	3,185
F3	67,406	2,143.90	16,081	2,999
M1	69,773	1,411	10,507	1,909
M2	45,376	1,736.20	14,749	2,419
M3	61,091	1,733.80	15,214	2,449
PM1	44,372	1,975.50	18,741	2,658
PM2	33,131	2,111.70	15,836	2,846
PM3	42,922	2,165.80	17,949	2,916
PF1	33,804	1,798.50	15,043	2,603
PF2	29,205	1,848.70	16,471	2,688
PF3	46,574	2,474.30	22,595	3,520

Table S3

ID number	Chromosome	type	group	Up/down	Gene
XM_006119029.2	NW_005854023.1	m5c	PM/F	+	ODF2
XM_006119029.2	NW_005854023.1	m6a	PM/F	+	ODF2
XM_006125703.3	NW_005855804.1	m5c	PM/F	+	NBR1
XM_006125722.2	NW_005855804.1	m5c	PF/M	+	TMUB2
XM_006125778.2	NW_005855804.1	m5c	PF/M	+	RUNDC1
XM_006126588.3	NW_005855937.1	m5c	PF/M	-	MIEF2
XM_006126614.3	NW_005855937.1	m5c	PF/M	+	GID4
XM_006137207.3	NW_005870997.1	m6a	PF/M	-	PACS2
XM_014571488.2	NW_005854023.1	m6a	PM/F	+	AK1
XM_014571506.2	NW_005854023.1	m5c	PM/F	+	ODF2
XM_014574896.2	NW_005855804.1	m5c	PF/M	+	VPS25
XM_014574898.2	NW_005855804.1	m5c	PF/M	+	RUNDC1
XM_014580679.2	NW_005870997.1	m6a	PF/M	-	PACS2
XM_014580680.2	NW_005870997.1	m6a	PM/F	+	PACS2
XM_014580682.2	NW_005870997.1	m6a	PF/M	-	PACS2
XM_014580682.2	NW_005870997.1	m6a	PM/F	+	PACS2
XM_025178154.1	NW_005859001.1	m6a	PF/M	-	UBE2O
XM_025178157.1	NW_005859001.1	m6a	PF/M	-	UBE2O
XM_025178157.1	NW_005859001.1	m6a	PM/F	-	UBE2O
XM_025178158.1	NW_005859001.1	m6a	PM/F	-	UBE2O
XM_025178158.1	NW_005859001.1	m6a	PF/M	-	UBE2O
XM_025179588.1	NW_005871042.1	m5c	PF/M	-	ABHD2
XM_025179588.1	NW_005871042.1	m5c	PM/F	-	ABHD2
XM_025181374.1	NW_005853326.1	m5c	PM/F	+	NDFIP2
XM_025181377.1	NW_005853326.1	m5c	PF/M	+	NDFIP2
XM_025185967.1	NW_005855804.1	m6a	PF/M	+	NBR1
XM_025185967.1	NW_005855804.1	m5c	PM/F	+	NBR1

Table S4

Sample	Sequence	Trans
ODF2-qF	AGCCGATGAGGTGACGGTAAA	XM_006119029.2
ODF2-qR	GCTCAATCCGCTGACGAAGAT	
PASC2-1-qF	GCTGGACGTGTTCATCGAGAA	XM_006137207.3
PASC2-1-qR	CCTTGCCAGTCCGAAGTGTTA	
AK1-qF	CTCCTCAGAATGGCGACAGAA	XM_014571488.2
AK1-qR	ATGGCGTCCCGCAACATATCA	
PASC2-2-qF	GCTGGACGTGTTCATCGAGAA	XM_014580679.2
PASC2-2-qR	TTGGGATCTGTAGGTGGTGCC	
PASC2-3-qF	ACCATTCTGGGCTACAAGACCTT	XM_014580680.2
PASC2-3-qR	CATCATCACTGGCTTCCTGCTC	
UBE20-qF	TAGGTCAGGTGGCTCGTGTTG	XM_025178154.1
UBE20-qR	GGCTCTTCTATTCTCGGATGGTC	
UBE20-qF	GCACCACCGACATTGTGATTC	XM_025178157.1
UBE20-qR	CACCCTGCACTTGTTCTTCTACC	
4085-f	GTTTGAAGTGCTGCTGGGAAG	
4085-r	TTCCCCGTATAAAGCCAGGG	
B-Actin-qF	TGTGCGTGACATCAAGGAAA	
B-Actin-qR	CACAGGATTCCATACCCAGG	
